# Paralytic Shellfish Toxins and Ocean Warming: Bioaccumulation and Ecotoxicological Responses in Juvenile Gilthead Seabream (*Sparus aurata*)

**DOI:** 10.3390/toxins11070408

**Published:** 2019-07-13

**Authors:** Vera Barbosa, Marta Santos, Patrícia Anacleto, Ana Luísa Maulvault, Pedro Pousão-Ferreira, Pedro Reis Costa, António Marques

**Affiliations:** 1 IPMA—Portuguese Institute for the Sea and Atmosphere, I.P., Av. Doutor Alfredo Magalhães Ramalho, nº 6, 1495-165 Algés, Portugal; 2 CIIMAR—Interdisciplinary Centre of Marine and Environmental Research, University of Porto, Terminal de Cruzeiros do Porto de Leixões, Avenida General Norton de Matos S/N, 4450-208 Matosinhos, Portugal; 3 MARE—Marine and Environmental Sciences Centre, Guia Marine Laboratory, Faculty of Sciences, University of Lisbon (FCUL), Av. Nossa Senhora do Cabo, 939, 2750-374 Cascais, Portugal; 4 IPMA—Portuguese Institute for the Ocean and Atmosphere, EPPO-Aquaculture Research Station, 8700-305 Olhão, Portugal; 5 CCMAR—Centre of Marine Sciences, University of Algarve, Campus of Gambelas, 8005-139 Faro, Portugal

**Keywords:** Paralytic shellfish toxin, warming, fish, seafood safety, ecotoxicological responses

## Abstract

Warmer seawater temperatures are expected to increase harmful algal blooms (HABs) occurrence, intensity, and distribution. Yet, the potential interactions between abiotic stressors and HABs are still poorly understood from ecological and seafood safety perspectives. The present study aimed to investigate, for the first time, the bioaccumulation/depuration mechanisms and ecotoxicological responses of juvenile gilthead seabream (*Sparus aurata*) exposed to paralytic shellfish toxins (PST) under different temperatures (18, 21, 24 °C). PST were detected in fish at the peak of the exposure period (day five, 0.22 µg g^−1^ N-sulfocarbamoylGonyautoxin-1-2 (C1 and C2), 0.08 µg g^−1^ Decarbamoylsaxitoxin (dcSTX) and 0.18 µg g^−1^ Gonyautoxin-5 (B1)), being rapidly eliminated (within the first 24 h of depuration), regardless of exposure temperature. Increased temperatures led to significantly higher PST contamination (275 µg STX eq. kg^−1^). During the trial, fish antioxidant enzyme activities (superoxide dismutase, SOD; catalase, CAT; glutathione S-transferase, GST) in both muscle and viscera were affected by temperature, whereas a significant induction of heat shock proteins (HSP70), Ubiquitin (Ub) activity (viscera), and lipid peroxidation (LPO; muscle) was observed under the combination of warming and PST exposure. The differential bioaccumulation and biomarker responses observed highlight the need to further understand the interactive effects between PST and abiotic stressors, to better estimate climate change impacts on HABs events, and to develop mitigation strategies to overcome the potential risks associated with seafood consumption.

## 1. Introduction

Harmful algae blooms (HABs) naturally occur under favorable environmental conditions, leading to the proliferation and/or aggregation of microalgae species containing high levels of toxic compounds, i.e., marine biotoxins [1]. The geographic distribution of toxic algae species has been associated with changes in local or regional eutrophication conditions, or due to large-scale climatic changes [2]. Indeed, coastal eutrophication and extreme climate events, such as El Niño, may promote favorable growing conditions (i.e., nutrient enriched waters) for the occurrence of toxic algal blooms, and therefore increased HAB events [1,2]. HABs are a major concern for marine ecosystems, as they can translate in several toxicological effects to the marine species that ingest them, being particularly deleterious to individuals in early life stages [3,4]. Moreover, HAB events have a great impact on human health, due to the consumption of contaminated seafood [1]. Filter-feeding organisms, such as bivalves, feed toxic microalgae and accumulate toxins they produce. Recently, other taxonomic groups higher up in the food chain (e.g., predatory fish, cephalopods, birds, and mammals) have been also pointed out as important vectors of marine biotoxins. Yet, so far, little attention has been paid to the transfer and toxicological mechanisms of marine toxins in these “emerging vector species” [5,6].

Marine biotoxins can be classified according to their solubility (i.e., hydrophilic or lipophilic), as well as their toxicological mode of action (i.e., paralytic shellfish poisoning, PSP; amnesic shellfish poisoning, ASP; diarrheic shellfish poisoning, DSP; neurotoxic shellfish poisoning, NSP; and ciguatera fish poisoning, CFP) [7]. Among the hydrophilic biotoxins are paralytic shellfish toxins (PST) and amnesic shellfish toxins (AST), whereas diarrheic shellfish toxins (DST), neurotoxic shellfish toxins (NST), and ciguatoxins (CTX) are lipophilic compounds [8]. Paralytic shellfish toxins (PST), including saxitoxin and saxitoxin-related compounds (STXs), are potent neurotoxins mainly produced by marine dinoflagellates that cause PSP [1,8]. PST neurotoxicity is due to their high affinity to bind to voltage-gated sodium channels, inhibiting the passage of sodium ion nerve cell membranes, and thus blocking neuronal and muscular activities [4,8]. As PST toxicity differs according to the binding affinity of each compound [7], carbamate toxins, including saxitoxin (STX), neosaxitoxin (NEO), and gonyautoxins (GTX1 and GTX4) have been considered the most toxic PSTs, followed by their decarbamoyl derivatives (dcSTX, dcGTX, and dcNEO), whereas N-sulfocarbamoyl toxins (e.g., B1 (GTX5), B2 (GTX6), and C1 and C2) are usually associated with lower toxicity [7,8].

Over the past decades, HABs have increased in frequency, intensity, and geographic distribution, mainly due to the increase in seawater temperatures that favors microalgae growth [9]. Indeed, worldwide, climate change is increasing seawater surface temperature (SST), and this trend is expected to worsen over the next decades, with SSTs increasing up to 5 °C in some regions [2,10]. Yet, both direct and indirect impacts of climate change effects in marine ecosystems, especially in the food-web system, are still unclear [9]. Understanding the way and extent to which abiotic variables can affect the occurrence/toxicity of HABs in seafood species will make it possible to anticipate how climate change drivers affect marine species from both an ecological and a seafood safety perspective. 

PST exposure [11] and climate change effects [12] are known to induce adverse effects on fish species, mainly in the antioxidant mechanism as a result of oxidative stress [13]. Induced oxidative stress may exert cytotoxic effects through the overproduction of reactive oxygen species (ROS), which are involved in cellular protective mechanisms, but at higher concentrations lead to deleterious effects in cellular proteins, lipids, and DNA [14]. Several biochemical assays can be used to evaluate the ecotoxicological responses induced by exposure to environmental contaminants and climate change stressors [12,14]. Within fish antioxidant machinery, catalase (CAT) and superoxide dismutase (SOD) are considered ROS scavengers with protective roles against ROS formation, while glutathione S-transferases (GST) plays a key role in organs’ second phase detoxification [15]. In addition, heat shock proteins (HSP) are mainly associated with cellular redox changes by temperature, and lipid peroxidation (LPO) is the ultimate degradation product of cellular injury [15]. Yet, such an approach has not been employed to evaluate the ecotoxicological effects of PST under warming.

Within this context, the present study aims to assess, for the first time, the effect of seawater temperature regimes on PST (C1 and C2, dcSTX and B1) bioaccumulation and depuration mechanisms in juvenile fish, as well as its ecotoxicological responses, following five days of dietary exposure to these toxins. Gilthead seabream (*Sparus aurata*) was selected as the biological model, since it is a predatory fish with high commercial value, widely produced in coastal areas of the eastern Atlantic and Mediterranean Sea [16]. Blue mussels (*Mytilus galloprovincialis*) constitute a natural prey of fish species inhabiting the Mediterranean region, such as *S. aurata*, and this bivalve species is a primary vector of PST in coastal areas [17]. Therefore, naturally-contaminated mussels were used as feed to expose juvenile seabream to PST. 

## 2. Results

### 2.1. Influence of Warming on PST Accumulation and Depuration

**Figure 1:**
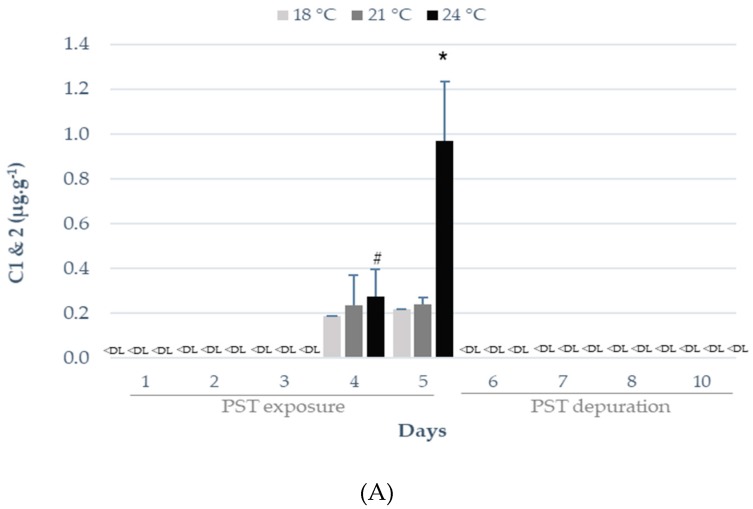

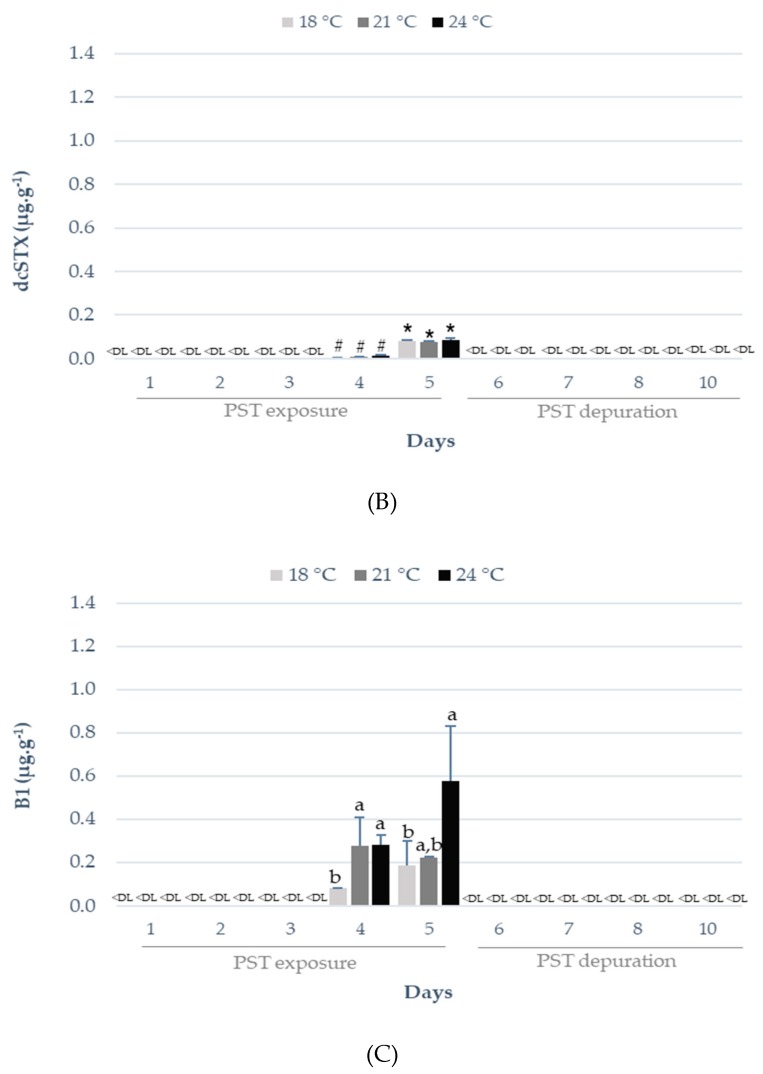
Effect of different temperature regimes (18 °C, 21 °C, and 24 °C) on the accumulation/depuration of paralytic shellfish toxins (PST) (µg g^−1^) in *Sparus aurata*: (**A**) N-sulfocarbamoylgonyautoxin-1 and -2 (C1 and C2), (**B**) decarbamoylsaxitoxin (dcSTX), (**C**) gonyautoxin-1 (B1), during the experimental period. Results are expressed as mean ± SD (*n* = 5). Different letters (a, b, c) indicate significant differences (*p* < 0.05) between temperatures, whereas the symbols (*, #) indicate significant differences (*p* < 0.05) between days. <DL = below detection limit.

PST were detected after four days of exposure (regardless of temperature regime), with the highest concentration being found on day five at 24 °C (0.97 μg g^−1^ C1 and C2, 0.57 μg g^−1^ B1 and 0.09 μg g^−1^ dcSTX) (Figure 1). The PST profile was limited to C1 and C2, dcSTX and B1 toxins analogues, matching the toxin profile of contaminated mussels’ hepatopancreas used as feed. C1, C2, and B1 toxins were the most abundant PST in seabream juvenile specimens (Figure 1A,C). Still, on day four, higher concentrations were observed for the B1 toxin (0.27 μg g^−1^; Figure 1C), whereas on day 5, higher concentrations were observed for C1 and C2 (0.97 μg g^−1^; Figure 1A). PST were not detected (levels below detection limit) during the depuration period (i.e., days 6–10), indicating a fast elimination rate in this fish species (Figure 1A–C).

On day four, higher temperatures (21 °C and 24 °C) significantly increased (*p* < 0.05) B1 toxin levels in seabream juveniles (Figure 1C), whereas on day five (i.e., maximum PST exposure), significantly higher concentrations of C1, C2, and B1 toxins were observed in fish exposed to the highest seawater temperature (i.e., 24 °C; *p* < 0.05; Figure 1A,C). In addition, warming significantly increased C1 and 2 concentration with time (day five > day four), while dcSTX concentration was significantly higher on day five, regardless of seawater temperature (Figure 1A,B). PST toxicity was calculated using the toxicity equivalency factors (TEFs) adopted for each toxin group [18]. Warming significantly increased (*p* < 0.05) PSP toxicity at the maximum exposure period, where the maximal toxicity of 275 ± 3 µg STX eq. kg^−1^ was reached on day five and 24 °C (Table 1). Moreover, PSP toxicity in seabream juveniles significantly increased from day four to day five at higher temperatures (21 °C and 24 °C; Table 1).

### 2.2. Influence of Warming and PST Exposure on Fish Ecotoxicological Responses

**Figure 2:**
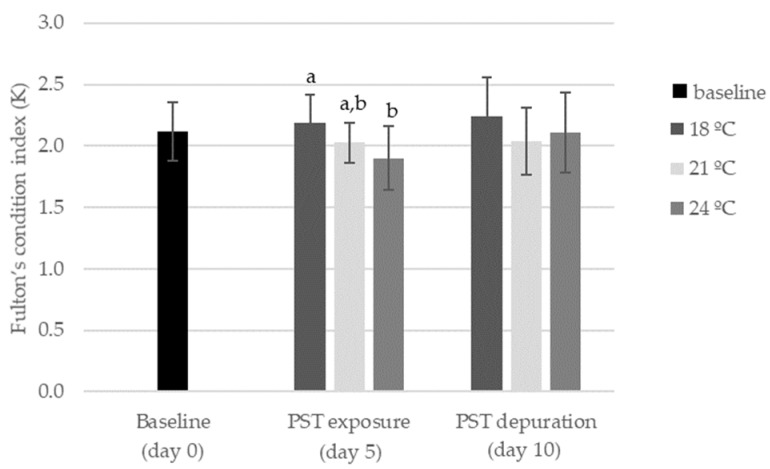
Fulton’s condition index (K) in *S. aurata* before PST exposure (day 0), after five days of exposure (day 5) and after five days of depuration (day 10) at different temperatures (mean ± SD; *n* = 5). Different letters (a, b) indicate significant differences (*p* < 0.05) between treatments (18 °C, 21 °C, and 24 °C) for each day.

No mortality or changes in fish behavior were observed during the experiment. The combined effect of PST exposure and warming significantly decreased (*p* < 0.05) animal condition, as a significantly lower Fulton’s condition index (K) was observed at 24 °C and on day five (PST exposure) (Figure 2). On the other hand, no significant differences (*p* < 0.05) were observed on day 10 (depuration), regardless of water temperature (Figure 2).

**Figure 3:**
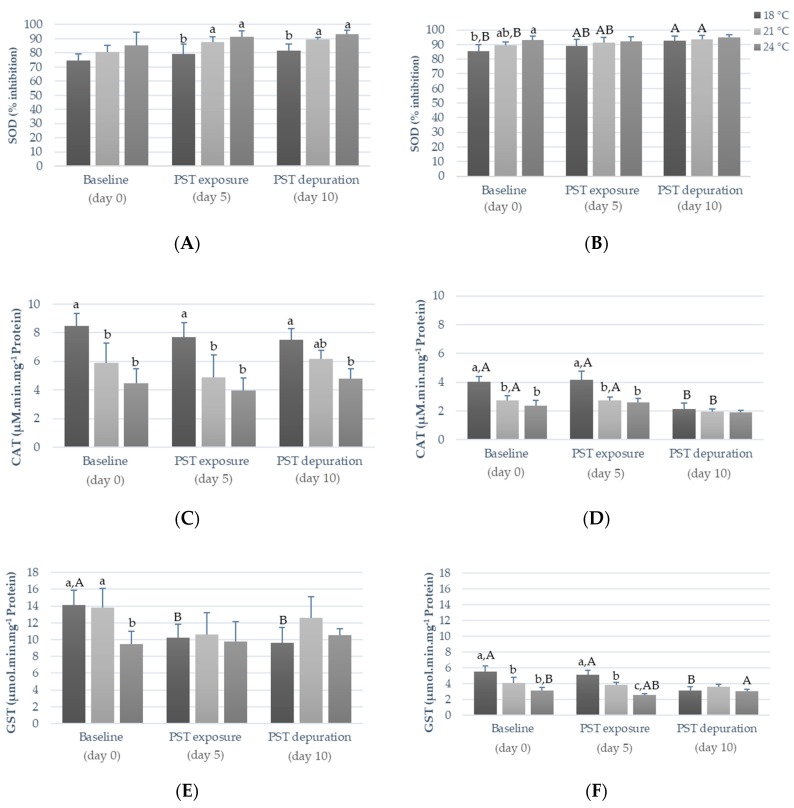
Anti-oxidant enzyme activities (SOD; CAT; GST) in the viscera (**A**,**C**,**E**) and muscle (**B**,**D**,**F**) of *S. aurata* before PST exposure (day 0), after five days of PST exposure (day 5) and after five days of depuration (day 10) at different temperatures (18 °C, 21 °C, and 24 °C). Results are expressed as mean ± SD (*n* = 5). Different letters (a, b, c) indicate significant differences between temperatures (*p* < 0.05), whereas (A, B) indicate significant differences between days. Abbreviations: CAT—catalase activity; SOD—superoxide dismutase inhibition; GST—glutathione S-transferase activity.

The levels of oxidative stress-related enzymes, including SOD, CAT, and GST activities, are presented in Figure 3. In fish viscera, warming (i.e., exposure to 21 °C and 24 °C) significantly inhibited (*p* < 0.05) SOD activity after PST exposure (day five), as well as during the depuration phase (day ten; *p* < 0.05; Figure 3A). Conversely, acclimation to warmer temperatures (before PST exposure, i.e., day zero) induced lower SOD activity in fish muscle (*p* < 0.05), such a trend was reversed after five days of concomitant exposure to PST, with SOD inhibition increasing in all treatments, regardless of temperature regime (Figure 3B). Moreover, SOD activity tended to decrease throughout time (i.e., day zero versus day ten) in the muscle of fish exposed to the lowest temperature regime (i.e., at 18 °C), but not in those exposed to warmer temperatures (Figure 3B). Warming (both temperatures) significantly reduced CAT activity in the viscera and muscle of fish, regardless of PST exposure (*p* < 0.05; Figure 2C,D), only except during the depuration period (day ten) in muscle (*p* < 0.05; Figure 3D). Noteworthy, throughout the experimental period, CAT activity decreased in the muscle of fish under the control temperature (18 °C), being significantly lower (*p* < 0.05) on day ten (PST depuration) compared to day zero (baseline) and day five (PST exposure; Figure 3D). At the beginning of the experiment (day zero, baseline), fish acclimated under warmer temperatures exhibited significantly lower GST activity (*p* < 0.05) in both tissues compared to those under the control temperature (Figure 3E,F). After five days of PST exposure (i.e., on day five), this trend was maintained in fish muscle (i.e., GST activity stayed significantly lower in fish exposed to warming, particularly to the highest temperature; Figure 3F) but not in the viscera, with PST exposure being responsible for a significant diminishment of GST activity in fish exposed to the control temperature (on both day five and day ten in viscera and on day ten in muscle; Figure 3E,F).

**Figure 4:**
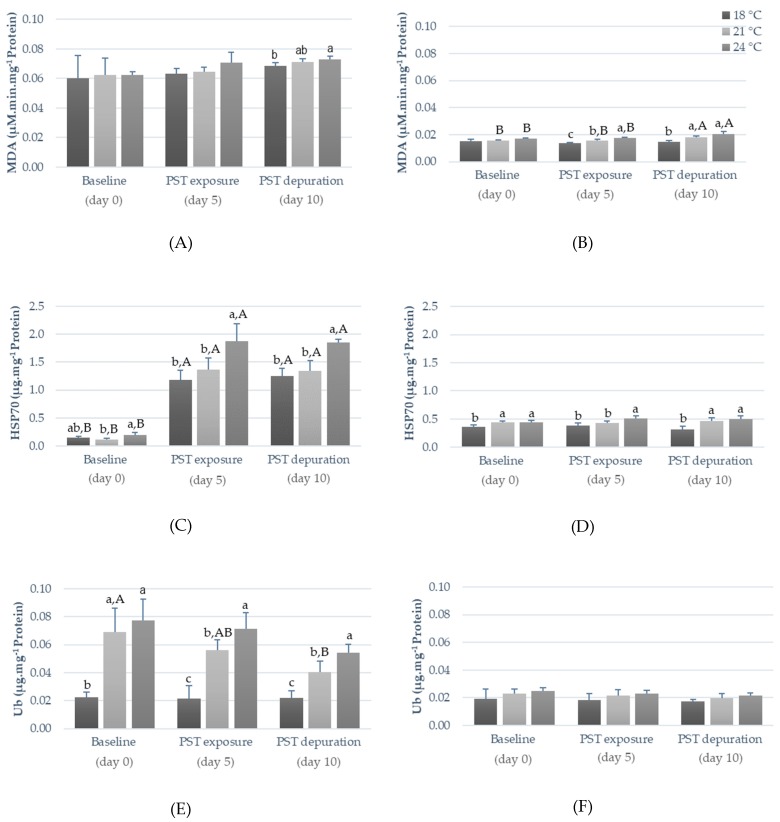
Lipid peroxidation (as MDA concentration), heat shock protein (HSP70) concentration, and ubiquitin concentration (Ub) in viscera (**A**,**C**,**E**) and muscle (**B**,**D**,**F**) of *S. aurata* before PST exposure (day 0), after five days of PST exposure (day 5) and after five days of depuration (day 10) at different temperatures (18 °C, 21 °C, and 24 °C). Results are expressed as mean ± SD (*n* = 5). Different letters (a, b, c) indicate significant differences between temperatures (*p* < 0.05), whereas (A, B) indicate significant differences between days. Abbreviations: MDA—malondialdehyde concentration.

Matching the overall inhibition of antioxidant enzyme activities promoted by warmer temperatures and/or PST exposure, LPO (measured as malondialdehyde (MDA) concentration) gradually increased over time in fish exposed at 21 °C and 24 °C, being significantly higher (*p* < 0.05) compared to the values observed in fish under 18 °C after five days of PST exposure (muscle) as well as after the PST depuration period (day 10; viscera and muscle; Figure 4A,B).

**Figure 5:**
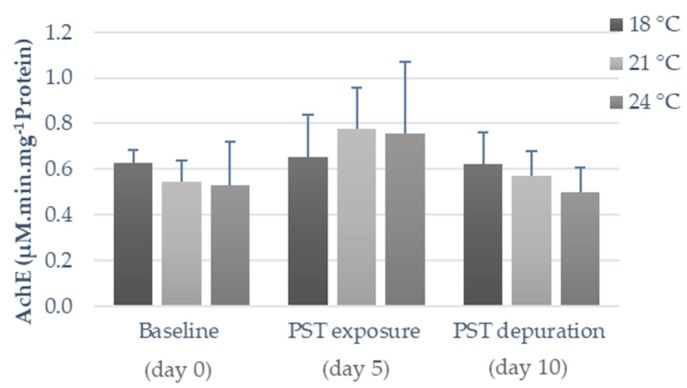
Acetylcholinesterase (AChE) activity in muscle tissue of *S. aurata* before PST exposure (day 0), after five days of PST exposure (day 5) and after five days of depuration (day 10) at different temperatures (18 °C, 21 °C, and 24 °C). Results are expressed as mean ± SD (*n* = 5).

Concerning heat shock response, HSP70 content in both fish tissues was significantly affected by temperature and PST exposure (Figure 4C,D). In fish viscera, PST exposure triggered a drastic increase in HSP70 proteins synthesis (*p* < 0.05), particularly at warmer temperatures (24 °C), a trend that was still observed even after the five days of the PST depuration period (Figure 3C). Conversely, in fish muscle, HSP70 levels did not seem to be significantly affected by PST exposure (i.e., no significant differences between days zero, five, and ten), whereas warmer temperatures, particularly 24 °C, increased the synthesis of these proteins for all sampling days (*p* < 0.05; Figure 4D). Warming (21 °C and 24 °C) significantly increased (*p* < 0.05) Ub protein synthesis in fish viscera, regardless of PST exposure, though a gradual decrease was observed throughout time in these two treatments (*p* < 0.05). Conversely, this tendency was not observed in the one simulating the control temperature (an increase between days zero, five, and ten was observed instead in fish exposed at 18 °C; Figure 4E). In comparison, fish muscle did not evidence significant differences in Ub contents (Figure 4F) nor in AChE activity (Figure 5).

## 3. Discussion

### 3.1. Influence of Warming on PST Accumulation and Depuration

In line with the present findings, low levels of toxins were observed in fish exposed to PST through feed, with toxin concentrations evidencing an increase with the exposure time (i.e., maximum concentration found by the end of the exposure period) [19,20]. Yet, contrasting with previous studies, PST profiles in fish (predators) are identical to the profiles of their prey (C1 and C2 > B1 > dcSTX). Only a few studies have focused on fish metabolism of PST. However, the differences previously reported in the toxin profiles of prey and predators suggest that PST biotransformation may also take place [19,21]. Nevertheless, the low levels detected, associated with identical elimination rates during uptake and depuration, may explain the absence of PST metabolization [22]. Several studies show that viscera are the primary organ for PST accumulation, but have also higher detoxification rates (excretion), which can be effectively accelerated in juvenile specimens that present rapid growth, and therefore faster metabolism [22].

In agreement with the present results, Costa et al. [19] and Kwong et al. [20] reported high toxin elimination in *Diplodus sargus* (B1 and dcSTX) and *Acanthopagrus schlegeli* (C1 and C2), suggesting that PST can be easily excreted by renal processes [23]. Interestingly, PSTs were not detected after the first 24 h of depuration. In contrast, previous reports showed decreased toxin concentrations during the first five days of depuration [19,21]. Such differences may be explained by different toxin profiles and model fish species. It is known that C2 and C1 toxin analogues, which were the predominant ones in our study, are less stable and easily undergo enzymatic hydrolysis, being rapidly eliminated via urine [19]. Moreover, warmer water temperatures lead to higher metabolic rates associated with the increase in fish energetic demands [24] and, consequently, higher excretion rates of the more soluble toxin analogues [22].

To the authors’ best knowledge, so far, the effect of warming on PST accumulation/depuration has only been assessed in bivalve species, i.e., oysters (*Crassostrea gigas* and *Saccostrea glomerata* [25]), sea scallops (*Placopecten magellanicus* [26]) and mussels (*Mytilus edulis* [26] and *Mytilus galloprovincialis* [27]), therefore hampering adequate comparisons of the present data (i.e., concerning a fish model species) with previous studies. However, while in *M. edulis* and *P. magellanicus* the effect of temperature on PST uptake was unclear [26], warmer temperatures significantly decreased PST concentrations in *S. glomerata*, diploid *C. gigas* [25], and *M. galloprovincialis* [27]. Contrarily, a previous study showed PST accumulation in the fish muscle of *Geophagus brasiliensis* during HABs, with slightly higher PST concentration in summer compared to spring and autumn, in a Brazilian reservoir [28]. Several studies demonstrated that warmer temperatures enhance organic compound accumulation in fish species (e.g., Hg in *Dicentrarchus labrax* [29], triclosan in *Diplodus sargus* [24]) via feed ingestion, as a result of enhanced fish metabolism and, therefore, increased feeding rates. Still, increased metabolic rates can also translate into increased compound metabolization and/or excretion [28], which may explain the present results. Despite the increased accumulation of PST in seabream at warmer temperatures, toxin concentrations remained below the current safety limits established for human consumption (800 μg STX eq. kg^−1^) [18]. Nevertheless, these limits were established to protect consumers [18], and therefore the potential toxic effect on fish welfare can be under or overestimated. Indeed, in terms of fish welfare it is worthwhile highlighting the ecotoxicological responses observed in juvenile gilthead seabream fish exposed to PST at warmer temperatures (e.g., decreased animal fitness). Yet, particularly noteworthy was the 1.4-fold increase in seabream toxicity with warming (24 °C), representing a 20% increase in PSP toxicity. These results strongly suggest that the higher toxin accumulation levels might be exacerbated if temperatures continue to increase to levels projected by the Intergovernmental Panel on Climate Change (IPCC) for the worst-case scenario. It is known that several bivalve species can convert N-sulfocarbamoyl toxins to their corresponding carbamate toxin (more toxic) under conditions of a high temperature and low pH [23]. Yet, such a process of conversion and/or metabolized in fish is still unclear and could not be detected in the current study.

Furthermore, in line with previous studies [30], the present results suggest that differences would be expected with adult seabream specimens. It is known that adult fish have higher rates of feed ingestion, meaning the ingestion of higher amounts of feed and, consequently, the ingestion of higher toxin concentrations. In addition, evidence of toxin biotransformation (the conversion of less potent and less stable toxins into more potent and stable ones) during digestion can occur by enzymes in adult fish, as well as toxin distribution through extravascular fluids to other organs revealing PST bioaccumulation within the food chain [30].

### 3.2. Influence of Warming and PST Exposure on Fish Biochemical Responses

In agreement with previous studies, an increased oxidative stress response was observed in both tissues due to warming and PST exposure in *S. aurata* [12,24,28,31,32]. Aquatic organisms’ antioxidant mechanisms are complex, and exposure to stressful environmental conditions can induce or inhibit antioxidant enzymes [28]. Generally, increased SOD and CAT activities are linked with warmer seawater temperatures, due to the enhancement of an organism’s metabolism [12,24,32], whereas SOD and CAT activities inhibition were observed after toxin and metal exposure [31,32,33]. The present results show that the combined exposure to PST and warming resulted in lower SOD activity in viscera in the beginning of the exposure trial, while in muscle, SOD activity was inhibited by warmer temperatures, regardless of PST exposure. It is known that the liver is the main organ for PST accumulation, being responsible for toxin biotransformation, redistribution to other tissues, and elimination [19]. Yet, both warming and PST exposure reduced CAT activity in viscera and muscle. A similar pattern was also found with xenobiotic compounds (e.g., endosulfan in *Channa punctatus* [34], MeHg in *Dicentrarchus labrax* [32], triclosan in *Diplodus sargus* [24], and STX in *Hoplias malabaricus* [35]). PST exposure may result in an intensive formation of reactive oxygen species (ROS), and such excessive substrate production (superoxide anion) may inhibit CAT activity [35]. As for GST, the activity inhibition in fish exposed to warmer temperatures (viscera and muscle) and PST (muscle) is in contradiction with previous studies carried out with MeHg [32] and other fish species, such as Atlantic salmon [36] and *Hoplias malabaricus* [35], where GST activity increased under stressful conditions and/or as a response to CAT activity inhibition [24,28,32,36]. PST metabolization mainly occurs in fish liver, where phase I and phase II reactions for the biotransformation of xenobiotics take place [20]. It has been hypothesized that C1, C2, and B1 analogues can enter directly phase II of biotransformation, yet so far GST induction has mainly been reported in fish exposed to PST carbamate analogues [20,21]. Furthermore, enzyme denaturation or cells’ inability to synthetize enzymes can occur when temperatures exceed threshold values [12]. On the other hand, GST activity inhibition indicates that the N-sulfocarbamoyl toxins (C1, C2, and B1) were highly hydrophilic and easily eliminated, not being necessary to promote PST analogue excretion through the conjugation of toxins with reduced glutathione (GHS; GST catalyze) [21]. Antioxidant enzymes are strongly species-dependent, as each species has different thermal tolerance limits and enzyme baseline levels [12,37]. Yet, the distinct trends observed show that increased temperatures affect fish antioxidant responses to PST exposure, in a tissue- and biomarker-specific way (e.g., enhanced SOD activity was observed in viscera, but not in muscle, and inhibited GST activity in muscle, but not in viscera). Interestingly, PST exposure strongly affected fish antioxidant machinery in muscle at 18 °C, since significantly lower SOD, CAT, and GST activities were observed after the PST depuration period, compared to the baseline, corroborating the fact that antioxidant enzyme activities are time-dependent [37]. 

The increase in LPO under warming and PST exposure indicate that membrane damage occurred over time (i.e., overall, higher values at the end of the trial compared to baseline values), particularly at warmer temperatures (possibly due to SOD, CAT, and GST inhibition), further corroborating the time-dependency of cells’ antioxidant scavengers and indicating that these enzymes were not able to totally prevent the oxidative damage induced by ROS, potentially leading to cell death [32]. The significantly higher MDA concentration observed in muscle after PST depuration at higher temperatures suggests that the combined effect of warming and PST exposure may lead to irreversible cell damage.

The increase in HSP70 is generally associated with rising temperatures, as well as with exposure to environmental contaminants [32,37]. Generally, HSP content gradually increases until reaching a maximum level and then it starts to decrease as thermal stress becomes more severe and protein synthesis mechanisms are led to exhaustion [12]. Noteworthy, HSP synthesis may be influenced by the baseline contents of each species and/or tissues, and by the synergistic effects of contaminants [32], explaining the significant increase in HSP content in viscera after PST exposure compared to non-exposed fish. In addition, the increased Ub levels in viscera after warming and PST exposure indicate that the synthesis of these proteins was triggered in response to stressful conditions, most likely as a result of an increased need for chaperoning and degradation by the proteasome of protein anomalies [37]. In what concerns muscular AChE, Clemente et al. [28] reported a significant increase after PST exposure at higher temperatures (summer). However, in the present work, both warming and PST exposure did not seem to affect AChE activity in fish muscle. 

## 4. Conclusions

The present study provides evidence that increased seawater temperatures facilitate PST bioaccumulation in juvenile seabream specimens, despite the possibility for these toxins to be rapidly depurated. Although warming promoted higher toxin accumulation by fish (1.4-fold increase), PSP toxicity levels remained below the current safety limits established for human consumption.

In terms of fish ecotoxicological responses, the co-exposure of warming with PST decreased animal fitness (K), and affected the biomarker responses of fish tissues, resulting in the inhibition of antioxidant scavengers (SOD, CAT, and GST) as well as in the enhancement of biomarkers involved in lipid (LPO) and protein (HSP70 and Ub) damage in cells. Yet, the impairment of fish antioxidant machinery under warming and PST exposure, alone or in co-exposure, suggests that ecotoxicological responses can only prevent oxidative stress to some extent, inducing cell damage, health problems and ultimately, fish mortality.

The different PST accumulation observed in fish exposed to warming conditions highlights the need to consider interactions between multiple stressors, especially linked with climate change scenarios (i.e., HABs, warming, and acidification), in future studies on toxin accumulation and elimination in commercial marine species, as well as for ecotoxicological responses. Such studies will allow the collection of more realistic information on the potential effects of climate change-related stressors on HABs toxicity potentially causing the impossibility to trade commercially valuable fish species.

## 5. Materials and Methods

### 5.1. Preparation of PST Contaminated Diet

Naturally contaminated and non-contaminated mussels (*Mytilus galloprovincialis*) were used to expose fish to PST through their diet. Contaminated *M. galloprovincialis* were collected in Aveiro Lagoon, NW Portuguese coast, during a bloom of *Gymnodinium catenatum* in late 2016. The presence of PST was confirmed by liquid chromatography, as previously described by Costa et al. [19]. Toxin composition included carbamate (STX, GTX2, and GTX3), N-sulfocarbamoyl (C1, C2, and B1) and decarbamoyl analogues (dcGTX2, dcGTX3, and dcSTX) (Table 2), and the PST toxicity measured was 27,232 µg STX eq. kg^−1^. Mussels hepatopancreas were dissected and freeze-dried at −50 °C, 10^−1^ atm of vacuum pressure, for 48 h (Power Dry 150 LL3000, Heto, Czech Republic), homogenized, and kept at −20 °C prior to the feeding experiments.

### 5.2. Experimental Design and Biological Sampling

**Figure 6:**
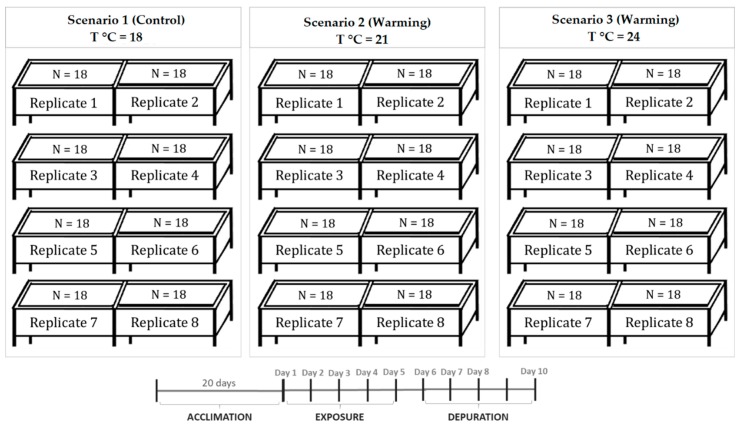
Experimental setup.

Juvenile specimens of *S. aurata* reared at the aquaculture pilot station of the Portuguese Institute for the Sea and Atmosphere (EPPO-IPMA, Olhão, Portugal), were maintained in 24 rectangular glass tanks (~50 L) in Guia Marine Laboratory (MARE-FCUL, Cascais, Portugal, with an independent water recirculation system (RAS), temperature and pH control (Profilux 3.1N, GHL, Germany), refrigeration system (Frimar, Fernando Ribeiro Lda, Portugal), protein skimmers (Reef SkimPro, TMC Iberia, Portugal), UV disinfection (Vecton 300, TMC Iberia, Portugal), and biological filtration (model FSBF 1500, TMC Iberia, Portugal). Seawater parameters were controlled daily through seawater renewal (25%) and by colorimetric tests (Tropic Marin, Montague, CA, USA). Ammonia and nitrites were kept below detectable levels, while nitrates were kept below 2.0 mg L^−1^. Seabream specimens were acclimated for 15 days in aerated seawater (dissolved O_2_ > 5 mg L^−1^) at 18 ± 0.5 °C, pH 8.0 ± 0.1 units, 35 ± 1.0 ‰ salinity, 12:12 h photoperiod and fed with 7% of the average body weight (b.w.), with a commercial fish diet manufactured by SPAROS, Lda (Olhão, Portugal). Detailed feed nutritional composition can be consulted in Table S1. Five days before initiating PST exposure, seawater temperature was slowly adjusted (1.0 ± 0.5 °C per day), until it reached 21 °C and 24 °C in the tanks, simulating warming conditions [10] and heat wave conditions [10,38], respectively. During this period, the commercial fish diet used to feed fish was replaced by lyophilized hepatopancreas of non-contaminated mussels (amount equivalent to 7.6% of fish b.w. with PST < DL), to allow fish to adapt to this new type of food. Three scenarios were carried out (*n* = 18 animals per replicate tank of treatment, i.e., total of 144 animals per treatment), simulating the current temperature conditions used in seabream rearing (18 °C), an increase in average seawater temperature simulating the warming conditions projected by the IPCC in the Mediterranean region (ΔT °C = +3 °C; RCP 8.5, IPCC, 2014), and seawater temperature increase simulating a heat wave event (ΔT °C = +6 °C; [39]) (Figure 6). During the five days of PST exposure, seabream juveniles were daily fed with PST contaminated mussels (lyophilized hepatopancreas; 7.6% b.w.; toxins’ profile presented in Table 2), and subsequently fed again with non-contaminated mussels (7.6% b.w.) during the five days of the depuration phase. During acclimation and the exposure trial, seawater abiotic parameters were checked daily and adjusted whenever needed.

For toxin extraction and quantification, 45 individuals were randomly collected 2 h after feeding, at days one, two, three, four, five (PST exposure), six, seven, eight, and ten (PST depuration). Fish were randomly collected from each treatment and euthanized by immersion in an overdosed MS222 solution (2000 mg L^−1^; Sigma-Aldrich, St. Louis, MO, USA) buffered with sodium bicarbonate (1 g of NaHCO_3_ to 1 g of MS222 to 1 L of seawater). In each temperature, 15 specimens were collected (*n* = five fish per replicate; three replicates) for each sampling day. Euthanized fish were measured (total length, TL, and weight, W) and whole body (without head) and immediately frozen at −20 °C until further analysis. For enzymatic and protein quantification assays, 15 seabream juveniles were randomly collected from each temperature, euthanized, and measured, at day zero (before PST exposure), day five (maximum PST exposure), and day ten (final day of depuration period). Fish were carefully dissected, and fish muscle and viscera tissues (i.e., liver, pancreas, and intestines) were collected and immediately frozen at −80 °C until further analysis. Details regarding fish biometry can be consulted in Table S2.

### 5.3. Toxins Extraction and Quantification

Toxins from whole fish homogenate were heat-extracted in 1% acetic acid, vortexed, and centrifuged (15,000× *g*) for 10 min. Extracts followed a solid-phase extraction (SPE) with an octadecyl bonded phase silica (Supelclean LC-18 SPE cartridge, 3 mL, Supelco, Bellefonte, PA, USA). Periodate and peroxide oxidations of PST were carried out and toxins were immediately quantified by high performance liquid chromatography with fluorescence detection (HPLC-FLD), based on the precolumn oxidation method developed by Lawrence and Niedzwiadek (2001) [39]. The HPLC-FLD equipment consisted of a Hewlett–Packard/Agilent Model 1290 Infinity quaternary pump, autosampler, column oven, and Model 1260 Infinity fluorescence detector. PST oxidation products were separated using a reverse-phase Supelcosil LC-18, 15 × 4.6, 5 µm column (Supelco, Bellefonte, PA, USA). The mobile phase gradient consisted of 0–5% B (0.1 M ammonium formate in 5% acetonitrile, pH 6) in the first 5 min, 5–70% B for the next 4 min, and back to 0% B in the next 2 min. Then, 100% mobile phase A (0.1 M ammonium formate, pH 6) was used for 3 min before the next injection. The flow rate was 1 mL min^−1^ and the detection wavelength set to 340 nm for excitation and 395 nm for emission. Instrumental limits of detection (S/N = 3) were 11 ng g^−1^ dcSTX, 12 ng g^−1^ STX, 12 ng g^−1^ B1, 19 ng g^−1^ for dcGTX2 and dcGTX3, and GTX2 and GTX3, 34 ng g^−1^ C1 and C2. Working standard solutions for calibration curves were prepared by the dilution of PST stock solutions in PST-free cleaned-up fish tissue extract. Certified calibration solutions for PST were purchased from the Certified Reference Materials Program of the Institute for Marine Biosciences, National Research Council, Canada (STX-e, NEO-b, GTX2-b and GTX3-b, GTX1-b and GTX4-b, dcSTX, dcGTX2 and dcGTX3, GTX5-b (B1), C1 and C2, and dcNEO-b).

### 5.4. Biochemical Assays

Fish tissues (muscle and viscera) were homogenized in ice-cold conditions with 1.5 mL of phosphate buffered saline (PBS; 140 mM NaCl, 3 mM KCl, 10 mM KH_2_PO_4_, pH = 7.40 ± 0.02; reagents from Sigma-Aldrich, Steinheim, Germany), using an Ultra-Turrax^®^ device (T25 digital, Ika, Germany) and centrifuged in 2 mL microtubes for 15 min at 10.000× *g* and 4 °C. Then, the supernatants were transferred to new microtubes, immediately frozen, and kept at −80 °C until further analysis. Seven molecular biomarkers were selected to assess the biological responses to PST exposure and warming at the tissue level. A summary of the selected biomarkers is presented in Table 3, with reference to the different methodologies used (further details regarding these methodologies have been previously described by Madeira et al. [12], Maulvault et al. [24], and Maulvault et al. [32]). All biochemical analyses were performed in triplicate and using reagents of pro analysis grade or higher. Total protein levels were quantified in each sample in order to enable the subsequent normalization of each biomarker (i.e., given in mg of protein; methodology based on the Bradford assay [40]). All methodologies were adapted to 96-well microplates, as previously reported by Maulvault et al. [33].

### 5.5. Animal Fitness Index (Fulton’s K index)

The Fulton’s K index was directly calculated from the biometric data to determine fish condition, according to the formula,

K = 100 × (W/TL^3^), (1)

where W is the total wet weight (g) and TL is total length (cm).

### 5.6. Statistical Analysis

Results were expressed as mean values ± standard deviation (SD). ANOVA assumptions of normality and homoscedasticity were tested through Kolmogorov–Smirnov and Levene tests, respectively. Data were log-transformed or square rooted transformed, whenever at least one of the ANOVA assumptions was not verified. To evaluate the presence of significant differences between whole organism PST accumulation and temperature, one-way ANOVA analysis was performed. In terms of biochemical biomarkers (CAT, SOD, GST, LPO, HSP70, Ub, and AChE) and fish condition (W, TL, and K), a two-way ANOVA was carried out, using tissue (viscera and muscle), temperature (i.e., 18 °C, 21 °C, and 24 °C) and treatment (Baseline (day zero), PST exposure (day five), and PST depuration (day ten)) as variables. Subsequently, post-hoc Tukey HSD tests were performed. Potential correlations between biomarker levels and the animal fitness index (Fulton’s K index) were performed by means of the Pearson’s correlation coefficient. Statistical analyses were performed at a significance level of 0.05, using STATISTICA™ software (Version 7.0, StatSoft Inc., Tulsa, OK, USA).

## Figures and Tables

**Table 1:** Toxicity (µg STX eq. kg^−1^) of *S. aurata* exposed via feed to PST at different temperatures and current EU limit [11] for paralytic shellfish poisoning (PSP) toxins.

Sampling Day	Temperature	Toxicity (µg STX eq. kg^−1^)	EC 853/2004^11^
Day 4	18 °C	74.7 ± 1.6	800 µg STX eq. kg^−1^
	21 °C	59.1 ± 8.3 ^#^	
	24 °C	67.1 ± 6.5 ^#^	
Day 5	18 °C	113.4 ± 17.2 ^b^	
	21 °C	154.1 ± 1.2 ^b,^*	
	24 °C	275.4 ± 3.0 ^a,^*	

Different letters (a, b) indicate significant differences (*p* < 0.05) between temperature (18 °C, 21 °C, and 24 °C) for the same day, whereas the symbols (*, #) indicate significant differences (*p* < 0.05) between days for the same temperature. Results are expressed as mean ± SD (*n* = 5).

**Table 2:** Toxin profile (mg kg^−1^) in mussels (*M. galloprovincialis*) hepatopancreas given as food to gilthead seabream (*S. aurata*).

Toxins Analogues	DW	WW
**dcGTX2 and dcGTX3**	4.6	1.1
**C1 and C2**	248.9	60.8
**dcSTX**	50.4	12.3
**GTX2 and GTX3**	1.5	0.4
**B1**	156.1	38.1
**STX**	0.6	0.1
**Total Toxicity** **(mg STX eq. kg^−1^)**	111.5	27.23

DW—dry weight; WW—wet weight. dcGTX2 and dcGTX3 (decarbamoylgonyautoxin-2 and -3); C1 and C2 (N-sulfocarbamoylgonyautoxin-2 and -3); dcSTX (decarbamoylsaxitoxin); GTX2 and GTX3 (gonyautoxin-2 and -3); B1 (gonyautoxin-1); STX (saxitoxin).

**Table 3:** Summary of selected molecular biomarkers and the corresponding methodologies used.

Molecular Biomarker	Ecotoxicological Response	Methodology Used	References
Superoxide dismutase (SOD)	Oxidative stress	Enzymatic assay	[12,24,32]
Catalase (CAT)	Oxidative stress	Enzymatic assay	[12,24,32]
Glutathione S-transferase (GST)	Oxidative stress and xenobiotic detoxification phase II	Enzymatic assay	[12,24,32]
Heat shock response (HSP70)	Chaperoning, heat shock response	Indirect ELISA	[24,32]
Ubiquitin (Ub)	Protein degradation and DNA repair	Direct ELISA	[24,32]
Lipid peroxidation (LPO)	Oxidative stress and cellular damage	Thiobarbituric acid reactive substances (TBARS) method	[12,24,32]
Acetylcholinesterase (AChE)	Neurotoxicity	Enzymatic assay	[24,32]

## Supplementary Materials

The following are available online at https://www.mdpi.com/2072-6651/11/7/408/s1, Table S1: Commercial feed WIN Fast composition, by SPAROS, Lda (Olhão, Portugal), Table S2: Total length (TL; cm) and weight (W; g) of sampled specimens of S. aurata (mean ± standard deviation; *n* = 15) during the experiment.
