# Supplementary material for: Paralytic Shellfish Toxins and Ocean Warming: Bioaccumulation and Ecotoxicological Responses in Juvenile Gilthead Seabream (Sparus aurata)
Source: Toxins (Basel). 2019 Jul 13;11(7):408. doi: 10.3390/toxins11070408 (PMC6669718; doi:10.3390/toxins11070408)
Supplement: Supplementary file 1 [file toxins-11-00408-s001.pdf]

Supplementary Materials: Paralytic Shellfish Toxins and Ocean Warming: Bioaccumulation and Ecotoxicological Responses in Juvenile Gilthead Seabream (*Sparus aurata*)

Vera Barbosa, Marta Santos, Patrícia Anacleto, Ana Luísa Maulvault, Pedro Pousão-Ferreira, Pedro Reis Costa and António Marques

Table S1. Commercial feed WIN Fast composition, by SPAROS, Lda (Olhão, Portugal).

**Ingredients**
Fishmeal, squid meal, krill meal, wheat gluten, fish protein concentrate, Fish oil
**Chemical composition**
Crude protein (60%), crude fat (19%), crude fibber (0.3%), crude ash (6%), phosphorus (2%), calcium (0.8%), sodium (0.5%)
Vitamin D3 (2900 IU), L-taurine (10 mg Kg-1), betaine (10 mg Kg-1)
Ferric sulphate (180 mg Kg-1), calcium iodate (6 mg Kg-1), copper sulphate (25 mg Kg-1), manganese oxide (35 mg Kg-1), zinc sulphate (50 mg Kg-1), sodium selenite (0.3 mg Kg-1)

Table S2. Total length (TL; cm) and weight (W; g) of sampled specimens of *S. aurata* (mean ± standard deviation; *n* = 15) during the experiment (days 1 to 5: PST exposure; days 6 to 10: PST depuration).

Treatments		day 0	day 1	day 2	day 3	day 4	day 5	day 6	day 7	day 8	day 10
TL	Baseline	2.233 ± 0.216									
	18 °C		2.29 ± 0.41	2.02 ± 0.23	2.18 ± 0.24	2.24 ± 0.37	2.29 ± 0.24	2.30 ± 0.31	2.39 ± 0.16	2.40 ± 0.23	2.17 ± 0.18
	21 °C		2.16 ± 0.18	2.04 ± 0.30	2.15 ± 0.24	2.27 ± 0.21	2.19 ± 0.23	2.11 ± 0.19	2.22 ± 0.26	2.27 ± 0.25	2.28 ± 0.26
	24 °C		2.11 ± 0.19	2.23 ± 0.30	2.11 ± 0.25	2.12 ± 0.22	2.25 ± 0.32	2.23 ± 0.26	2.30 ± 0.26	2.26 ± 0.42	2.28 ± 0.30
W	Baseline	0.240 ± 0.069									
	18 °C		0.26 ± 0.13	0.20 ± 0.07	0.22 ± 0.09	0.26 ± 0.14	0.27 ± 0.09	0.26 ± 0.10	0.28 ± 0.07	0.29 ± 0.08	0.23 ± 0.06
	21 °C		0.22 ± 0.07	0.22 ± 0.11	0.21 ± 0.08	0.26 ± 0.11	0.22 ± 0.07	0.17 ± 0.05	0.21 ± 0.10	0.22 ± 0.08	0.25 ± 0.09
	24 °C		0.20 ± 0.05	0.25 ± 0.11	0.20 ± 0.07	0.20 ± 0.06	0.24 ± 0.12	0.23 ± 0.09	0.25 ± 0.11	0.24 ± 0.15	0.26 ± 0.11
